# Clonal versus non-clonal milkweeds (*Asclepias* spp.) respond differently to stem damage, affecting oviposition by monarch butterflies

**DOI:** 10.7717/peerj.10296

**Published:** 2020-11-03

**Authors:** Elise He, Anurag A. Agrawal

**Affiliations:** Ecology and Evolutionary Biology, Cornell University, Ithaca, NY, USA

**Keywords:** Butterfly conservation, Clonal plants, Plant-insect interactions

## Abstract

**Background:**

Oviposition decisions are critical to the fitness of herbivorous insects and are often impacted by the availability and condition of host plants. Monarch butterflies (*Danaus plexippus*) rely on milkweeds (*Asclepias* spp.) for egg-laying and as food for larvae. Previous work has shown that monarchs prefer to oviposit on recently regrown plant tissues (after removal of above-ground biomass) while larvae grow poorly on plants previously damaged by insects. We hypothesized that these effects may depend on the life-history strategy of plants, as clonal and non-clonal milkweed species differ in resource allocation and defense strategies.

**Methodology/Principal Findings:**

We first confirmed butterfly preference for regrown tissue in a field survey of paired mowed and unmowed plots of the common milkweed *A. syriaca*. We then experimentally studied the effects of plant damage (comparing undamaged controls to plants clipped and regrown, or damaged by insects) on oviposition choice, larval performance, and leaf quality of two closely related clonal and non-clonal species pairs: (1) *A. syriaca* and *A. tuberosa*, and (2) *A. verticillata* and *A. incarnata*. Clonal and non-clonal species displayed different responses to plant damage, impacting the proportions of eggs laid on plants. Clonal species had similar mean proportions of eggs on regrown and control plants (≈35–40% each), but fewer on insect-damaged plants (≈20%). Meanwhile non-clonal species had similar oviposition on insect-damaged and control plants (20–30% each) but more eggs on regrown plants (40–60%). Trait analyses showed reduced defenses in regrown plants and we found some evidence, although variable, for negative effects of insect damage on subsequent larval performance.

**Conclusions/Significance:**

Overall, non-clonal species are more susceptible and preferred by monarch butterflies following clipping, while clonal species show tolerance to clipping and induced defense to insect herbivory. These results have implications for monarch conservation strategies that involve milkweed habitat management by mowing. More generally, plant life-history may mediate growth and defense strategies, explaining species-level variation in responses to different types of damage.

## Introduction

Oviposition choices by herbivorous insects strongly impact fitness. Specifically, the suitability of an oviposition site influences hatching success, larval performance, and susceptibility to enemies (Resetarits, 1996). Consequently, as suggested by optimal oviposition theory and the preference-performance hypothesis, the most suitable plants for offspring survival and performance should be preferred for oviposition if they are available (Jaenike, 1978; Gripenberg et al., 2010). Like many lepidopterans, monarch butterflies (*Danaus plexippus)* are host specific—they lay eggs exclusively on one group of plants (primarily milkweeds in the genus *Asclepias*) and their larvae exclusively feed on these plants. Following recent reports of declining monarch populations in Eastern North America (Agrawal & Inamine, 2018; Brower et al., 2012; Vidal & Rendón-Salinas, 2014), some researchers have suggested strategically removing above-ground portions of milkweed to induce regrowth as a management strategy to increase monarch numbers because regrowth foliage is highly preferred by monarchs. Specifically, Fischer et al. (2015), Knight et al. (2019), and Haan & Landis (2019b) experimentally showed that mowing *Asclepias syriaca* plots at appropriate times in the summer can increase densities of eggs naturally laid by monarchs. Similarly, Baum & Sharber (2012) found that summer-prescribed fire on *A. viridis* increased egg and larval densities in late summer and early fall. Besides extending the growing season of milkweed by providing fresh regrowth when plants would otherwise be senescing (Alcock, Brower & Williams, 2016) and reducing predator numbers during the weeks needed to recolonize regrowing milkweed (Haan & Landis, 2019a, 2019b), removing above-ground tissue including apical buds of milkweed may also alter leaf quality traits in ways that are favorable for monarchs.

Many previous studies have found that plant damage can influence leaf quality for herbivorous insects (Karban & Baldwin, 1997). Generally, insect damage has been found to increase defense (Chen, 2008; Green & Ryan, 1972; Van Zandt & Agrawal, 2004), while damaging plants in a way that removes apical buds often increases leaf quality for herbivorous insects and grazers (Agrawal & Spiller, 2004; Danell & Huss-Danell, 1985; Nykänen & Koricheva, 2004). For example, leaf damage and defoliation of mountain birches induced phenolic compounds (Tuomi et al., 1988) and reduced insect performance (Haukioja, 1982), while removing apical buds on the same species induced ramets to produce larger leaves with higher water content, improving insect performance (Senn & Haukioja, 1994). Opposing plant responses to leaf feeding vs. damage that removes apical meristems (including mowing) may be driven by differential physiological responses and may be quite general (Karban & Niiho, 1995; Karban & Baldwin, 1997). For monarchs on milkweed, insect herbivory typically increases latex production and stronger resistance to larvae (Agrawal et al., 2015; Van Zandt & Agrawal, 2004), while regrown tissues after apical bud or shoot removal are preferred for monarch oviposition (Zalucki & Kitching, 1982), suggesting higher leaf quality or lower defenses in those plants (Agrawal, 2017).

There are over 140 species of *Asclepias* of varying life-history strategies, including clonal and non-clonal species, that inhabit diverse habitats across North America (Agrawal et al., 2015). Non-clonal species primarily regrow from shoot removal by reallocating energy and activating dormant buds on existing stems. Clonal plants have additional meristems stored underground and thus have additional strategies including translocation and reallocation of resources in rhizomes along with activation of new stems, increasing their ability to regrow after disturbance. This makes clonal plants potentially more tolerant to shoot removal events such as grazing (Schmid et al., 1988; Liu et al., 2007) and abiotic disturbances (Fischer et al., 2015; Schmid et al., 1988). Theory on plant growth and defense predicts that resource allocation to growth trades off with defense, suggesting that clonal plants, which have higher regrowth capacities, may have lower defenses and higher leaf quality for herbivores (Herms & Mattson, 1992; Jong & Meijden, 2000). The resource availability hypothesis predicts that although growth-dominated species may be less constitutively defended, they may show higher levels of plasticity, including induced defense (Coley, Bryant & Chapin, 1985). Previous studies found that clonal reproduction repeatedly evolved in *Asclepias* species associated with greater investment in root biomass and lower leaf toxin concentrations, suggesting this trade-off may impact plant-herbivore interactions (Pellissier et al., 2016). Given that monarch butterflies have oviposition preferences for specific milkweed species (Haribal & Renwick, 1998; Jones & Agrawal, 2019; Ladner & Altizer, 2005; Pocius et al., 2018; Zalucki, Oyeyele & Vowles, 1989), we predicted that oviposition decisions may be impacted by plant life-history and previous damage, both which alter resource allocation within the plant.

Here we first confirmed monarch preference for clipped and regrown tissue in a field survey of mowed and unmowed patches of common milkweed, *A. syriaca*. We then tested the effect of two types of plant damage (stem clipping vs. leaf herbivory by monarch caterpillars) on adult female monarch oviposition preferences by conducting choice experiments in field cages. We conducted this work on two phylogenetic pairs of species, each with a clonal and non-clonal representative, respectively: *A. syriaca* and *A. tuberosa*, and *A. verticillata* and *A. incarnata*. Other plant traits may accompany the differences in clonality we observed, and we consider these part of the life-history effects we sought to test. We predicted that (1) insect herbivory would induce plant resistance, (2) clipping to mimic mowing would induce susceptibility, and (3) the strength of these effects would be dependent on the extent of clonality of the species. In particular, non-clonal species were predicted to induce stronger resistance to insect damage and greater susceptibility to clipping compared to the same treatments in clonal species. These results were also expected to be reflected in assays of plant traits and larval growth.

## Materials and Methods

### Field survey

In 2019, we surveyed seven paired mowed and unmowed patches (blocks) of *A. syriaca* at Cornell University’s Dunlop Meadow Natural Area (Brooktondale, NY, USA: 42.386, −76.395). Within each pair, one block of milkweeds had been mowed regularly prior to the survey, ~25 cm off the ground twice annually during the growing season, and the other was not mowed. In 2019, mowing occurred on July 29. We surveyed five milkweed ramets randomly, each separated by at least two meters, within each block on two dates: once before mowing on July 23 and once after mowing on August 17 when mowed plants had regrown. On July 23, milkweed plants in blocks that were regularly mowed were 36% shorter than milkweeds in blocks that were not mowed during the growing season. After plants had regrown on August 17, milkweeds in mowed blocks were 82% shorter than milkweeds in unmowed blocks. On each survey date, we measured the height in cm, number of leaves, number of monarch eggs, and number of monarch feeding circles (circular holes on leaves that are indicative of initial chewing by first instar larvae) on each plant. We analyzed the number of monarch eggs and monarch feeding circles across treatments separately for each sampling date, as different plants were randomly sampled each time, using generalized linear mixed models with negative binomial distributions in the *lme4* package in R. For July 23 (before mowing), mowing treatment, height, and number of leaves were included as fixed effects. For August 17 (after mowing), only mowing was included as a predictor as no other factors were predictive. Pair and block nested within pair were included as random effects. We tested for significance of fixed effects using likelihood ratio chi-square tests.

### Monarch oviposition choice

We studied two pairs of closely related clonal and non-clonal milkweed species—(1) *A. syriaca* and *A. tuberosa*, and (2) *A. verticillata* and *A. incarnata* (Table 1). *A. syriaca* and *A. tuberosa* are closely related in the Temperate North American clade and *A. verticillata* and *A. incarnata* are closely related in the Incarnatae clade (Fishbein et al., 2011). Seeds of all plant species were washed in 10% bleach, scarified, cold stratified for 4 days at 4 °C, then incubated for 3 days at 28 °C. Seedlings were planted in 10 cm diameter pots with a peat-based soil (Lamberts LM1) and grown in a growth chamber at 14 h day:light cycle, 28 °C during the day and 26 °C at night (400 microeinsteins of light). Plants were watered when the soil was dry, approximately every 2–3 days, and fertilized once per week (N:P:K 21:5:20, 150 ppm N (μg/g)). A total of 5 weeks after planting, we transplanted the plants into 15 cm diameter pots. For each species, three treatments—clipped and regrown, insect-damage, and undamaged control—were randomly assigned. Regrowth treatments were imposed by clipping just above the cotyledons near soil level to simulate mowing 5 weeks before the oviposition trials. We specifically cut above cotyledons to allow non-clonal species to activate dormant buds and regrow. At the time of oviposition trials, the majority of clipped plants had regrown to their previous heights or taller. For the insect-damage treatments, we placed newly-hatched monarch caterpillars on each plant 5 days before the oviposition trials and allowed them to feed until removal right before the trials. We employed this scheme because previous work demonstrated the strongest induced responses to monarch herbivory occur 5 days after initial feeding (Agrawal, Patrick & Hastings, 2014), while effects of clipping require more time for new tissue growth. When experiments were conducted 8 weeks after initial planting, insect-damaged and control plants were ~1.7–3.2 times taller than clipped and regrown plants, varying depending on species.

**Table 1:** Characteristics of the four *Asclepias* species used in monarch oviposition choice experiments.

Species	Clade	Habitat	Clonal potential	Clonality
*A. syriaca*	Temperate North American	North eastern open fields	31	Clonal
*A. tuberosa*	Temperate North American	Dry fields and open woodlands	0.3	Non-clonal
*A. verticillata*	Incarnatae	Midwestern prairie	1.4	Clonal
*A. incarnata*	Incarnatae	Eastern wetlands	0.4	Non-clonal

**Note:**

Shown are each species’ clade (Fishbein et al., 2011), natural habitats (Woodson, 1954), and clonal potential defined as root buds per plant after ~45 days of growth (Pellissier et al., 2016).

To conduct oviposition choice experiments, we transported the plants to a field site and used a paired-cage design. We had 16 paired trials for *A. syriaca* and *A. tuberosa* and 13 for *A. verticillata* and *A. incarnata*. In each trial, a single mated female monarch reared from a laboratory colony was placed in a 1 m^3^ cage in a plowed field in Dryden, NY, USA. In each cage, the monarch was first exposed to a set of three plants of a single species (clipped and regrown, insect-damaged, and control). After a sufficient amount of time for at least 10 eggs to be laid in the cage, the set of plants was removed, and the monarch was then exposed to a new set of plants (the three treatments, but of the paired species). The lengths of trials varied as sets were replaced when at least 10 eggs were laid or after 3 h if fewer than 10 eggs were laid. At the end of each trial, the eggs on each plant were counted and removed.

We analyzed differences in oviposition using a generalized linear mixed effects model in the *lme4* package in R for each species pair separately. The proportion of eggs laid was the response variable, and species, treatment, and the interaction between species and treatment were included as fixed effects. Plant height was included as a covariate. Species within cage was included as a random effect. Significance of fixed effects was determined using Type III analysis of variance with Satterthwaite’s method. Pairwise comparisons between treatments were conducted using Tukey’s HSD using the *emmeans* package.

To evaluate larval performance, we placed one newly hatched caterpillar on each plant after the oviposition trial and caterpillars were allowed to feed for 5 days. Caterpillars were then removed, frozen, and weighed (*N* ≥ 8 per treatment per host species), and masses were compared using a linear model in R (with species, treatment, and height as fixed effects). The number of lost caterpillars (plant abandonment or mortality) at the time of removal was counted and examined using a general linear model with a binomial distribution using the *lme4* package. The number of lost caterpillars was the response variable and species, treatment, height, and the interaction between species and treatment were included as fixed effects. Analysis was done using Type II Wald Chi-square tests using the Anova function in the *car* package.

On a subset of each species by treatment combination (*N* = 5), we measured and analyzed leaf quality traits by evaluating latex exudation and trichome density. To analyze latex exudation, the tip of the youngest fully expanded leaf was cut and the latex exuded was absorbed onto a pre-weighed 1 cm diameter filter paper. To measure trichome density, we took circular leaf disks using a 0.25 in diameter puncher on the same youngest fully expanded leaf next to where it had been cut for latex exudation. Images of fresh leaf punches were taken using a camera attached to a microscope and we counted the number of trichomes on each punch using ImageJ. These leaf quality measurements across treatments were analyzed using one-way ANOVA and Tukey’s HSD post-hoc tests.

## Results

### Field survey

**Figure 1:**
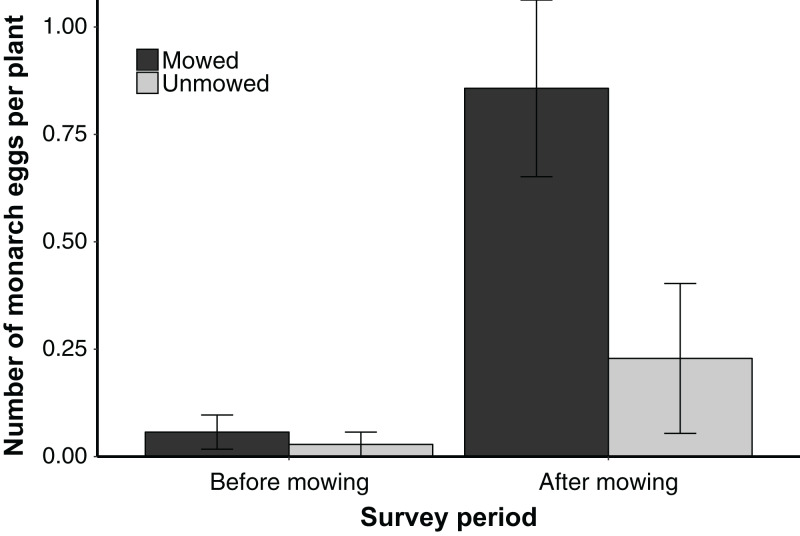
Mean ± SE number of monarch eggs found on individual mowed and unmowed *A. syriaca* plants in a field survey before mowing (July 23) and after mowing (August 17). Mowing occurred on July 29.

To confirm monarch preference for regrown tissue, a field survey was conducted on mowed and unmowed plots with abundant *A. syriaca*. Before mowing on July 23, there was no difference in number of monarch eggs (Table S1, LRT = 0.122, *p* = 0.727). Because very few eggs were observed (three in total), we also sampled numbers of initial monarch feeding circles; similarly, we found no difference in their numbers (mean ± SE circles per plant, mowed 3.60 ± 0.92, unmowed 3.57 ± 0.94, LRT = 0.184, *p* = 0.667). A total of 19 days after mowing, mowed plants that resprouted had >3 times more monarch eggs than unmowed plants (Table S1, LRT = 5.429, *p* = 0.020, Fig. 1).

### Monarch oviposition choice

**Figure 2:**
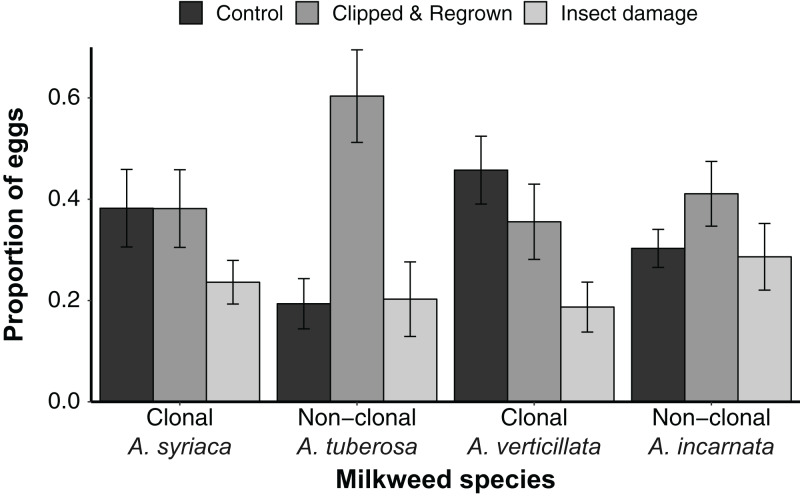
Mean ± SE proportion of eggs laid on different treatments for each milkweed species during oviposition trials. Clonal species show a greater (negative) effect of insect damage while non-clonal species show a stronger (positive) effect of apical clipping compared to undamaged controls.

Oviposition choice experiments were conducted on single plant species, but were paired such that members of the clonal and non-clonal pairs were sequentially tested with the same butterfly. For *A. syriaca* and *A. tuberosa*, the proportion of eggs laid on each plant in the set was impacted by treatment, and differentially between the two species (Fig. 2; Table S2, species by treatment interaction *F*_2,90_ = 4.323, *p* = 0.016). When clonal *A. syriaca* was analyzed separately, regrown and control plants had the same proportion of eggs on average (Fig. 2, ~38%), while insect-damaged plants had substantially fewer (24%); nonetheless, the treatment effect was not significant in this one-way analysis (*F*_2,43_ = 1.5783, *p* = 0.218). Treatment had a significant effect for non-clonal *A. tuberosa*, with clipped and regrown plants having about three times more eggs than either control or insect-damaged plants (Fig. 2, *F*_2,44_ = 4.209, *p* = 0.021). The difference was significant between regrown plants and control plants (Tukey HSD, *p* = 0.023) and between regrown plants and insect-damaged plants (Tukey HSD, *p* = 0.035).

The patterns of differential monarch oviposition for the second clonal—non-clonal milkweed pair, *A. verticillata and A. incarnata*, were remarkably similar to that of the first pair (Fig. 2). We again found evidence for a treatment by species interaction predicting egg laying, although the difference was marginal (Fig. 2; Table S2, species × treatment: *F*_2,72_ = 3.014, *p* = 0.057). In single species analyses, the effect of treatment was significant only for clonal *A. verticillata* (*F*_2,34_ = 1.578, *p* = 0.014), with insect-damaged plants having 47% fewer eggs than clipped and regrown plants and 59% fewer eggs than control plants on average (control vs. insect-damaged plants, Tukey HSD, *p* = 0.024). Treatment had no significant effect on the proportion of eggs on non-clonal *A. incarnata* plants alone (there was a significant effect of plant height, *F*_2,34_ = 7.858, *p* = 0.008), although clipped and regrown plants had ~38% more eggs than either insect-damaged or control plants.

### Larval performance

**Figure 3:**
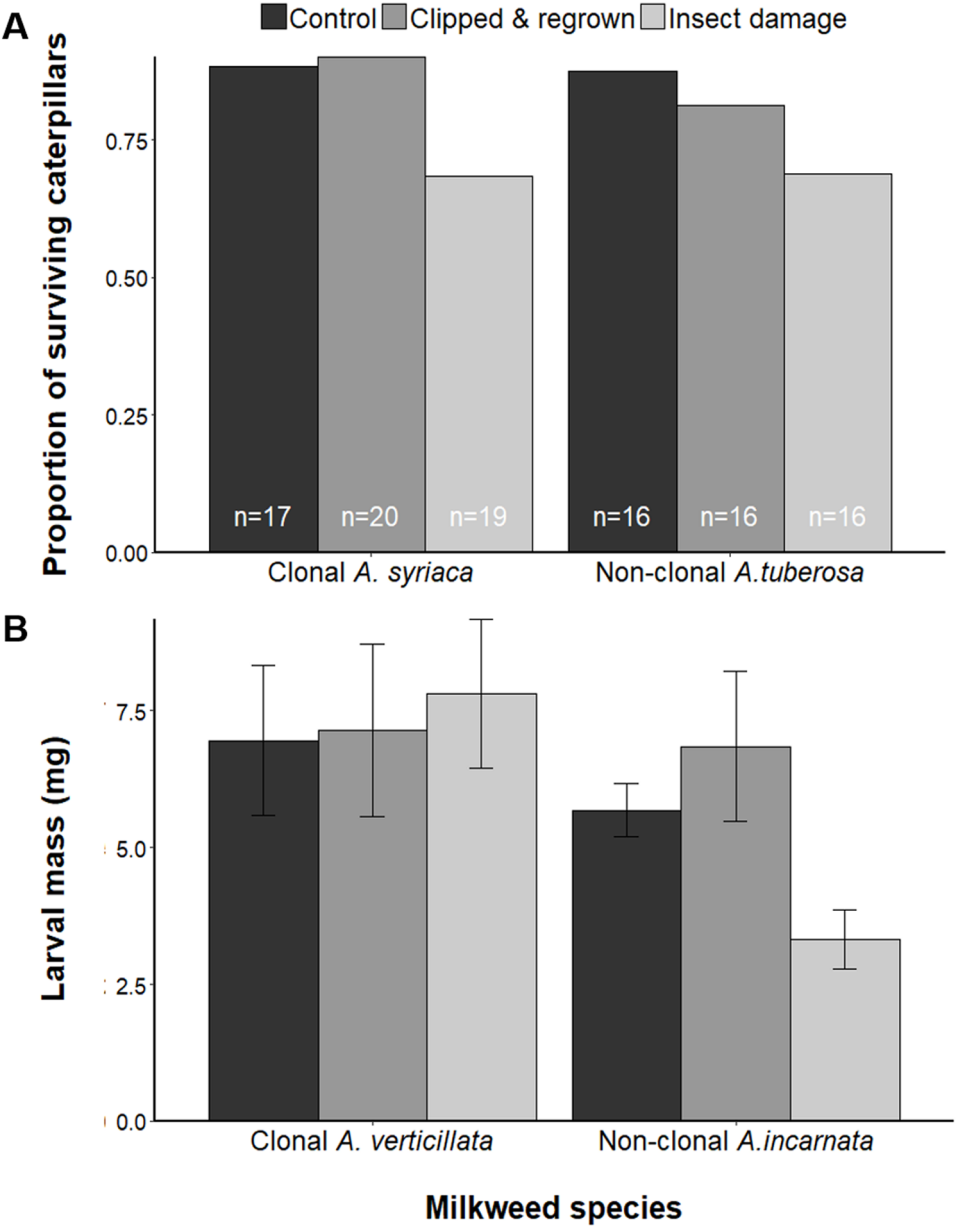
Effects of plant treatments on monarch larval performance. (A) Proportion of surviving larvae on each treatment of *A. syriaca* and *A. tuberosa*. We assume all larvae that had abandoned the plant did not survive. Bars are labeled with the total number of replicates for each treatment. For *A. syriaca* and *A. tuberosa*, there was no effect on larval mass (not shown). (B) Mean ± SE mass of larvae on each treatment for *A. verticillata* and *A. incarnata* (there was no effect on the proportion of surviving caterpillars).

While species and treatment affected oviposition, there were no overall effects on larval mass in either clonal and non-clonal species pair (Table S3). However, for both species of the *A. syriaca* and *A. tuberosa* pair, ~3 times more larvae disappeared (via plant abandonment or mortality) on insect-damaged plants than either regrown or control plants (Table S4, *p* = 0.087), causing 15–24% lower survival rates on insect-damaged plants (Fig. 3A). Within the second pair of species, treatment had a significant effect only on non-clonal *A. incarnata*, with larvae raised on regrown plants having two times the mass of larvae raised on insect-damaged plants (Fig. 3B, *F*_2,27_ = 4.004, *p* = 0.030).

### Trait comparisons

Latex exudation was measured only for *A. syriaca* as exudation was negligible in the other three species. For *A. syriaca*, latex mass was significantly affected by treatment (*F*_2,12_ = 4.359, *p* = 0.038), increasing by >3-fold in insect-damaged compared to clipped and regrown plants (Tukey HSD, *p* = 0.030; insect-damage 7.12 ± 1.75, clipped and regrown 2.29 ± 0.42, undamaged controls 4.90 ± 0.88).

Overall, variation in trichome densities among treatments showed similar trends across species. For *A. syriaca*, treatment had a strong effect on trichome density (*F*_2,12_ = 9.825, *p* = 0.003). Specifically, clipping reduced trichome density by about half compared to controls (Tukey HSD, *p* = 0.005, Table S5) and insect-damaged plants (Tukey HSD, *p* = 0.008, Table S5). Clipped and regrown *A. tuberosa* plants similarly had fewer trichomes compared to control and insect-damaged plants, although the treatment effect was non-significant (*F*_2,12_ = 3.112, *p* = 0.082, Table S5). As with the milkweeds of the first pair, damage treatments significantly affected trichome densities of *A. incarnata* plants. Clipped and regrown plants had lower trichome densities compared to the other treatments (*F*_2,12_ = 4.844, *p* = 0.029, Table S5). As the only exception, there was no effect of treatments on trichome densities of *A. verticillata* (*F*_2,12_ = 0.571, *p* = 0.579, Table S5).

## Discussion

Across the field survey and experiments, monarchs showed an overall increased preference toward regrown tissue of clipped plants and decreased preference toward insect-damaged plants, supporting previous studies which found that shoot removal (including mowing, clipping, and apical bud damage) induces susceptibility, while insect damage induces defenses (Green & Ryan, 1972; Karban & Niiho, 1995; Karban & Baldwin, 1997; Van Zandt & Agrawal, 2004; Chen, 2008). As predicted, the strength of these effects depended on the life-history strategy (clonality) of the species. On non-clonal milkweed species, the effect of clipping was strongly positive for oviposition choice. Meanwhile, on clonal milkweed species, insect damage treatment had the strongest (negative) effect on oviposition choice. This divergence may be caused by non-clonal species having lower capacities for regrowth after disturbance and are thus heavily affected by shoot and apical bud removal (Schmid et al., 1988; Liu et al., 2007). At the same time, non-clonal species were less impacted by insect damage, perhaps because of a slower growth rate and investment in constitutive defense. These effects may be general across *Asclepias* of varying life-histories, as tolerance (i.e., the ability to regrow) and resistance (i.e., having traits that reduce herbivore performance or preference) may be alternate defense strategies (Van der Meijden, Wijn & Verkaar, 1988; Karban & Baldwin, 1997; Strauss & Agrawal, 1999; Agrawal & Fishbein, 2008). Indeed, Agrawal et al. (2015) found that more phylogenetically derived *Asclepias* species tended to have enhanced clonal growth potential with increased tolerance to herbivory from the ability to sprout new ramets, while resistance traits such cardenolides and latex have declined with diversification. In our study, clonal species displayed not only strong tolerance but also inducibility, as oviposition decreased following insect attack. This may be because fast growing clonal species are overall less well defended, but have the capacity for plasticity (Coley, Bryant & Chapin, 1985; Mooney et al., 2010). In a study of clonal *Trifolium repens*, insect damage increased susceptibility in mature ramets while it induced defense in young ramets (Gómez et al., 2008). We used young plants with singular ramets, where tolerance strategies may not be strong enough for protection (Boege et al., 2007). Future work on clonal species should consider changes in allocation to growth and defense associated with plant ontogeny (Martínková et al., 2020).

While our field survey confirmed oviposition preference for regrown tissue, it also displayed a significant effect of clipping on a clonal species, *A. syriaca*, inconsistent with our experimental results. This may have been caused by increased apparency of regrown milkweed in mowed plots, as surrounding vegetation was also mowed (and very short). In unmowed plots, *A. syriaca* was surrounded by dense, tall vegetation. Another possible explanation is that mowing in field plots may have decreased total milkweed stem density, as higher mowing frequencies during the growing season are correlated with lower total number of stems (Dee & Baum, 2019). Indeed, some effects of clipping on oviposition have been shown to be dependent on plant density (Knight et al., 2019). It is also possible that in a field of established non-clonal milkweeds, the effect of clipping may be similarly magnified due to density reduction, although the lack of multiple ramets may increase priority and allocation to regrowth. Finally, in our experimental cage trials, all clipped plants were juvenile and cut above the cotyledons. In the field, mowing of mature plants may be a stronger inducer, activating new clonal stems, than clipping above the cotyledon (Paige, 1994).

Larval performance measured using caterpillar mass, unlike oviposition choice experiments, showed few differences between different species and treatments. Our analysis of larval performance suffered from low sample sizes, as many larvae had disappeared before they could be weighed. Nonetheless, previous studies of monarchs have also found a lack of support for the preference-performance hypothesis. Ladner & Altizer (2005) found that mean larval performance did not correspond to oviposition choices among four *Asclepias* species. Similarly, Jones & Agrawal (2019) found highest oviposition preference for a species associated with poor larval growth and low sequestration of plant toxins. In *A. syriaca* patches, Haan & Landis (2019b) found no significant changes in egg and larval survival after mowing despite increased oviposition. One possibility for the lack of a preference-performance link is that beyond leaf quality traits, oviposition stimulants such as flavonoid glycosides (Haribal & Renwick, 1996) and other plant volatiles (Bergström et al., 1994) may have an effect on oviposition. Here we measured latex exudation and trichome density, however a more comprehensive study with more plant trait measures is needed to clarify relationships between plant traits, oviposition choices and larval performance. Although trichomes are certainly deterrent to monarchs (caterpillars shave them on high density leaves), the responses we observed (reduction of trichomes on clipped plants) was consistent across species and likely associated with a growth enhancement (Agrawal, 2017). Furthermore, to fully assess larval performance, future work should assess effects of different types of plant damage on predation of monarch larvae. In a study of common milkweed in Michigan (USA), the abundance of monarch predators decreased following mowing, but direct links between mowing and the predator-prey interaction are not yet clear (Haan & Landis, 2019b).

### Implications for monarch butterfly conservation

The results of our study have implications for habitat management and monarch conservation. Previous studies have indicated that *A. syriaca* (clonal) and *A. viridis* (non-clonal) milkweed patches can be mowed or burned to increase eggs densities (Baum & Sharber, 2012; Fischer et al., 2015; Knight et al., 2019). Our results suggest that it may be relevant to consider the life-history strategy of the plant species being disturbed. Specifically, positive effects of plant clipping for monarchs may be highest on non-clonal species, although such species tend to be at lower stem-densities than clonal species. Future comparative work in natural populations will be needed to address the effects of clipping on monarch populations feeding on clonal vs. non-clonal milkweeds.

Any management strategy should be considered in conjunction with species preferences, as many studies have shown monarchs prefer ovipositing on particular milkweed species (Haribal & Renwick, 1998; Jones & Agrawal, 2019; Ladner & Altizer, 2005; Pocius et al., 2018; Zalucki, Oyeyele & Vowles, 1989). As different milkweed species often co-occur in the same field, these factors can interact to influence oviposition choices, and the strength of these factors may be patch-dependent. For example, Knight et al. (2019) found that while mowing in the summer generally increases monarch egg laying on mowed plots of *A. syriaca*, there were no effects on oviposition preferences in low-density patches (≤11 milkweeds/m^2^). Additionally, if there is no preference-performance linkage, the effectiveness of mowing programs may need to be re-evaluated.

Lastly, while mowing programs may increase egg densities, it is unclear whether the larvae produced will be able to contribute to the overall population regardless of whether larval performance corresponds to oviposition choices. The mowing programs proposed by Fischer et al. (2015) and Knight et al. (2019) induce freshly regrown tissues preferred by monarchs and extend their breeding season, but it is uncertain whether the new generation of monarchs that emerge can successfully join the migration to Mexico. As expressed by Alcock, Brower & Williams (2016), problems with this strategy include the replacement of senescing milkweed late in summer, which is important for stimulating the southern migration (Batalden & Oberhauser, 2015). Overall, the connection between increased egg densities and population-level effects needs substantial investigation.

### Conclusion: a general model for plant clonality, defense, and responsiveness to damage

Clonality in plants is generally seen as an adaptive response to environmental conditions (Fischer & Van Kleunen, 2001; Van Groenendael et al., 1996), specifically to spatial and temporal variability as clonal traits allow rapid recovery from frequent disturbance and seasonal inactivity (Suzuki & Stuefer, 1999). Across herbaceous plants, clonality increases with higher latitudes and greater seasonality (Ye et al., 2014). Clonal milkweed species have been found to inhabit colder and drier regions, where there may be lower herbivore pressure but higher climatic variability and relatively higher abiotic stresses such as trampling or fire (Pellissier et al., 2016). Conversely, resistance traits are often associated with environments with high herbivory (Karban & Baldwin, 1997; Chen, 2008) or warmer, tropical climates, where there is higher herbivore pressure (Pellissier et al., 2016). In our study, we found a weaker effect of abiotic disturbance (clipping) for clonal compared to non-clonal milkweed species. These effects may be general and apply to other systems—tolerance through clonality and regrowth protects plants against abiotic disturbances, where herbivore pressure may typically be low or unpredictable. Conversely, non-clonal plants may favor defenses overall, but respond to clipping by prioritizing growth at the cost of susceptibility to herbivores. A framework linking plant clonality and responses to damage therefore depends on the type of damage (abiotic disturbance or herbivory) that is most prevalent in the environment. We advocate future work that melds comparisons of closely related species with differing life-history strategies and incorporates both abiotic disturbance and insect attack.

## Supplemental Information

10.7717/peerj.10296/supp-1 Supplemental Information 1 Results of likelihood ratio tests of models estimating monarch egg counts in a field population of *Asclepias syriaca* on July 23 and Aug 17. Pair and block nested within pair were included as random effects.

10.7717/peerj.10296/supp-2 Supplemental Information 2 ANOVA estimating the effects of species, treatment, and their interaction on the proportion of eggs oviposited by monarch butterflies. Analyses were conducted separately for the two phylogenetic pairs of clonal and non-clonal milkweeds.

10.7717/peerj.10296/supp-3 Supplemental Information 3 ANOVA estimating the effects of species, treatment, plant height, and the interaction between species and treatment on mass of larvae. Analyses were conducted separately for the two phylogenetic pairs of clonal and non-clonal milkweeds.

10.7717/peerj.10296/supp-4 Supplemental Information 4 Chi-square analysis of deviance of binomial model estimating the number of missing larvae by the end of the experiment (*A. syriaca and A. tuberosa)*..

10.7717/peerj.10296/supp-5 Supplemental Information 5 Mean ± SE trichome densities (number of trichomes/mm^2^) on the four milkweed species compared in this study.

10.7717/peerj.10296/supp-6 Supplemental Information 6 Raw data.

## Additional Information and Declarations

### Data Availability

The following information was supplied regarding data availability: Raw data is available in the Supplemental Files.
