# Supplementary material for: Clonal versus non-clonal milkweeds (Asclepias spp.) respond differently to stem damage, affecting oviposition by monarch butterflies
Source: PeerJ. 2020 Nov 3;8:e10296. doi: 10.7717/peerj.10296 (PMC7646301; doi:10.7717/peerj.10296)
Supplement: Supplemental Information 1 — Pair and block nested within pair were included as random effects. [file peerj-08-10296-s001.docx]

**Table S1:** Results of likelihood ratio tests of models estimating monarch egg counts in a field population of *Asclepias syriaca* on July 23 and Aug 17. Pair and block nested within pair were included as random effects.

| Date | Fixed effect | df | LRT | P (Chi-square) |
| --- | --- | --- | --- | --- |
| July 23 | Mowing treatment | 1 | 0.122 | 0.727 |
|  | Height | 1 | 0.654 | 0.419 |
|  | Leaves | 1 | 0.709 | 0.400 |
| Aug 17 | Mowing treatment | 1 | 5.429 | 0.020 |
