# Supplementary material for: Clonal versus non-clonal milkweeds (Asclepias spp.) respond differently to stem damage, affecting oviposition by monarch butterflies
Source: PeerJ. 2020 Nov 3;8:e10296. doi: 10.7717/peerj.10296 (PMC7646301; doi:10.7717/peerj.10296)
Supplement: Supplemental Information 2 — Analyses were conducted separately for the two phylogenetic pairs of clonal and non-clonal milkweeds. [file peerj-08-10296-s002.docx]

**Table S2:** ANOVA estimating the effects of species, treatment, and their interaction on the proportion of eggs oviposited by monarch butterflies. Analyses were conducted separately for the two phylogenetic pairs of clonal and non-clonal milkweeds.

|  | Fixed effect | DF | F | p |
| --- | --- | --- | --- | --- |
| *A. syriaca*  & *A. tuberosa* | Species | 1, 90 | 0 | 1 |
|  | Treatment | 2, 90 | 9.125 | 0.001 |
|  | Species X Treatment | 2, 90 | 4.323 | 0.016 |
|  |  |  |  |  |
| *A. verticillata* & *A. incarnata* | Species | 1, 72 | 0 | 1 |
|  | Treatment | 2, 72 | 4.655 | 0.014 |
|  | Species X Treatment | 2, 72 | 3.014 | 0.057 |
