# Supplementary material for: Clonal versus non-clonal milkweeds (Asclepias spp.) respond differently to stem damage, affecting oviposition by monarch butterflies
Source: PeerJ. 2020 Nov 3;8:e10296. doi: 10.7717/peerj.10296 (PMC7646301; doi:10.7717/peerj.10296)
Supplement: Supplemental Information 3 — Analyses were conducted separately for the two phylogenetic pairs of clonal and non-clonal milkweeds. [file peerj-08-10296-s003.docx]

**Table S3:** ANOVA estimating the effects of species, treatment, plant height, and the interaction between species and treatment on mass of larvae. Analyses were conducted separately for the two phylogenetic pairs of clonal and non-clonal milkweeds.

|  | Fixed effect | DF | F-value | p-value |
| --- | --- | --- | --- | --- |
| *A. syriaca*  & *A. tuberosa* | Species | 1, 69 | 2.455 | 0.078 |
|  | Treatment | 2, 69 | 0.959 | 0.254 |
|  | Height | 1, 69 | 0.773 | 0.428 |
|  | Species X Treatment | 2, 69 | 0.004 | 0.996 |
|  |  |  |  |  |
| *A. verticillata* & *A. incarnata* | Species | 1, 49 | 0.203 | 0.039 |
|  | Treatment | 2, 49 | 1.892 | 0.580 |
|  | Height | 1, 49 | 0.626 | 0.298 |
|  | Species X Treatment | 2, 49 | 1.442 | 0.170 |
