# Supplementary material for: Clonal versus non-clonal milkweeds (Asclepias spp.) respond differently to stem damage, affecting oviposition by monarch butterflies
Source: PeerJ. 2020 Nov 3;8:e10296. doi: 10.7717/peerj.10296 (PMC7646301; doi:10.7717/peerj.10296)
Supplement: Supplemental Information 4 [file peerj-08-10296-s004.docx]

**Table S4:** Chi-square analysis of deviance of binomial model estimating the number of missing larvae by the end of the experiment (*A. syriaca and A. tuberosa).*

| Fixed effect | Df | Chi-sq | p-value |
| --- | --- | --- | --- |
| Species | 1 | 0.172 | 0.679 |
| Treatment | 2 | 4.890 | 0.087 |
