# Supplementary material for: Clonal versus non-clonal milkweeds (Asclepias spp.) respond differently to stem damage, affecting oviposition by monarch butterflies
Source: PeerJ. 2020 Nov 3;8:e10296. doi: 10.7717/peerj.10296 (PMC7646301; doi:10.7717/peerj.10296)
Supplement: Supplemental Information 5 [file peerj-08-10296-s005.docx]

**Table S5:** Mean ± SE trichome densities (number of trichomes/mm^2^) on the four milkweed species compared in this study.

|  | Clipped and regrown | Control | Insect damage |
| --- | --- | --- | --- |
| *A. syriaca* | 13.24 ± 3.16 | 26.17 ± 4.86 | 26.12 ± 7.62 |
| *A. tuberosa* | 3.56 ± 1.46, | 4.27 ± 1.14 | 6.89 ± 3.37 |
| *A. verticillata* | 4.62 ± 1.89 | 4.31 ± 1.12 | 5.71 ± 3.07 |
| *A. incarnata* | 2.88 ± 0.37 | 4.47 ± 1.11 | 4.28 ± 0.98 |
